# Revisiting Dynamic Processes and Relaxation Mechanisms in a Heterocyclic Glass-Former: Direct Observation of a Transient State

**DOI:** 10.1021/acs.jpcb.3c06727

**Published:** 2024-02-21

**Authors:** Andrzej Nowok, Hubert Hellwig, Mateusz Dulski, Maria Książek, Joachim Kusz, Piotr Kuś, Sebastian Pawlus

**Affiliations:** † Department of Experimental Physics, Wrocław University of Science and Technology, Wybrzeże Wyspiańskiego 27, Wrocław 50-370, Poland; ‡ Laboratoire National des Champs Magnétiques Intenses, EMFL, CNRS UPR 3228, Université Toulouse, Université Toulouse 3, INSA-T, Toulouse 31400, France; § Center for Integrated Technology and Organic Synthesis (CiTOS), MolSys Research Unit, University of Liège, B6a, Room 3/19, Allée Du Six Août 13, Liège, Sart Tilman 4000, Belgium; ∥ Faculty of Science and Technology, Institute of Materials Engineering, University of Silesia in Katowice, 75 Pułku Piechoty 1A, Chorzów 41-500, Poland; ⊥ August Chełkowski Institute of Physics, University of Silesia in Katowice, 75 Pułku Piechoty 1, 41-500 Chorzów, Poland; # Institute of Chemistry, University of Silesia in Katowice, Szkolna 9, Katowice 40-003, Poland

## Abstract

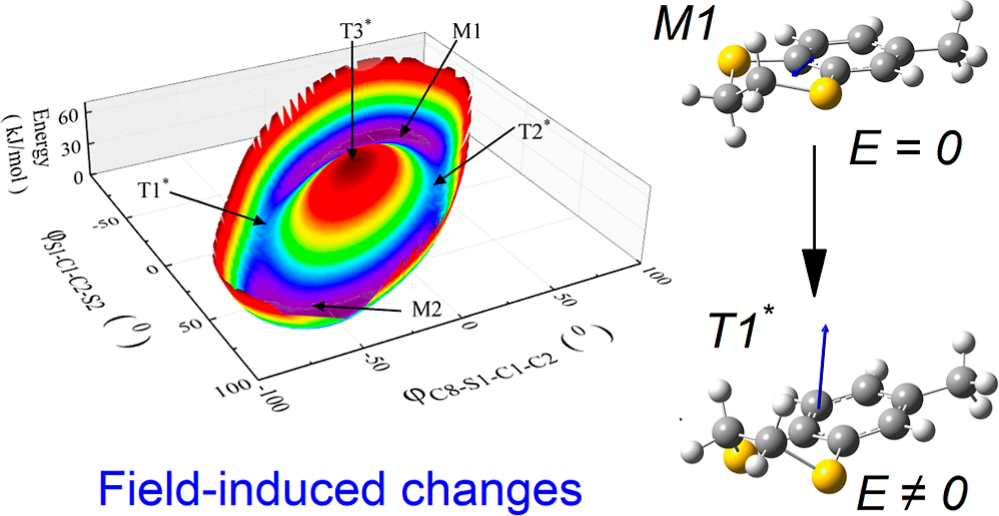

Despite decades of studies, a clear understanding of near-*T*_*g*_ phenomena remains challenging for glass-forming systems. This review delves into the intricate molecular dynamics of the small, heterocyclic thioether, 6-methyl-2,3-dihydro-1,4-benzodithiine (MeBzS_2_), with a particular focus on its near-*T*_*g*_ cold crystallization and relaxation mechanisms. Investigating isothermal crystallization kinetics at various temperatures reveals a significant interplay between its molecular dynamics and recrystallization from a supercooled liquid. We also identify two independent interconversion paths between energetically privileged conformers, characterized by strained transition states. We demonstrate that these spatial transformations induce substantial alterations in the dipole moment orientation and magnitude. Our investigation also extends to the complex salt PdCl_2_(MeBzS_2_), where we observe the transient conformers directly, revealing a direct relationship between their abundance and the local or macroscopic electric field. The initially energetically privileged isomers in an undisturbed system become less favored in the presence of an external electric field or ions, resulting even in an unexpected inversion of states. Consequently, we confirm the intramolecular character of secondary relaxation in MeBzS_2_ and its mechanism related to conformational changes within the heterocyclic ring. The research is based on the combination of broadband dielectric spectroscopy, X-ray diffraction, and quantum density functional theory calculations.

## Introduction

It has been known for centuries that a wide range of liquids and melted materials, including organic nonpolymeric compounds, can undergo supercooling well below their typical freezing temperatures, ultimately forming a disordered glassy state.^1,2^ This process, commonly called vitrification, encompasses many intricate physical phenomena that remain incompletely understood.^3,4^ Rapidly increasing viscosity, dramatic slowing down of molecular motion (both translational and rotational) from microseconds to hundreds of seconds, intermolecular self-organization into clusters, cold crystallization from supercooled liquid state, and intramolecular conformational changes are only several examples of the physicochemical processes occurring near glass transition temperature (*T*_*g*_).^4−10^ Among these phenomena, interconversion between conformers has attracted particular research interest.^11−14^

Conformational changes in organic molecules are considered an essential factor in crystallization.^15−18^ For example, comprehensive investigations into a collection of more than 400 acylanilides have revealed a clear correlation between an increasing abundance of energetically similar conformers, lengthened crystallization time, and a diminished propensity for crystallization.^15^ The mounting challenges in nucleation and further the crystal growth arise due to the necessity of selecting the appropriate conformer from a vast array of unsuitable ones that do not align with the crystal lattice, which is mainly hindered when the energy barrier related to conformational interconversion exceeds 10 kcal mol^–1^.^16−18^ Apart from the vitrification-facilitating role, conformational diversity may introduce misalignment of molecules in the liquid phase, leading to increased free volume between them and lowered *T*_*g*_.^19−22^ Finally, conformational alterations in organic glass-formers serve as an important source of intramolecular secondary relaxation processes in their dielectric response, which can be monitored by the broadband dielectric spectroscopy (BDS) technique.^5^ These dielectric processes, manifesting as step-like anomalies and loss peaks in the real and imaginary parts of the complex dielectric permittivity, occur in both the liquid and glassy phases and remain invariant to pressure changes.^5,23^

**Figure 1:**
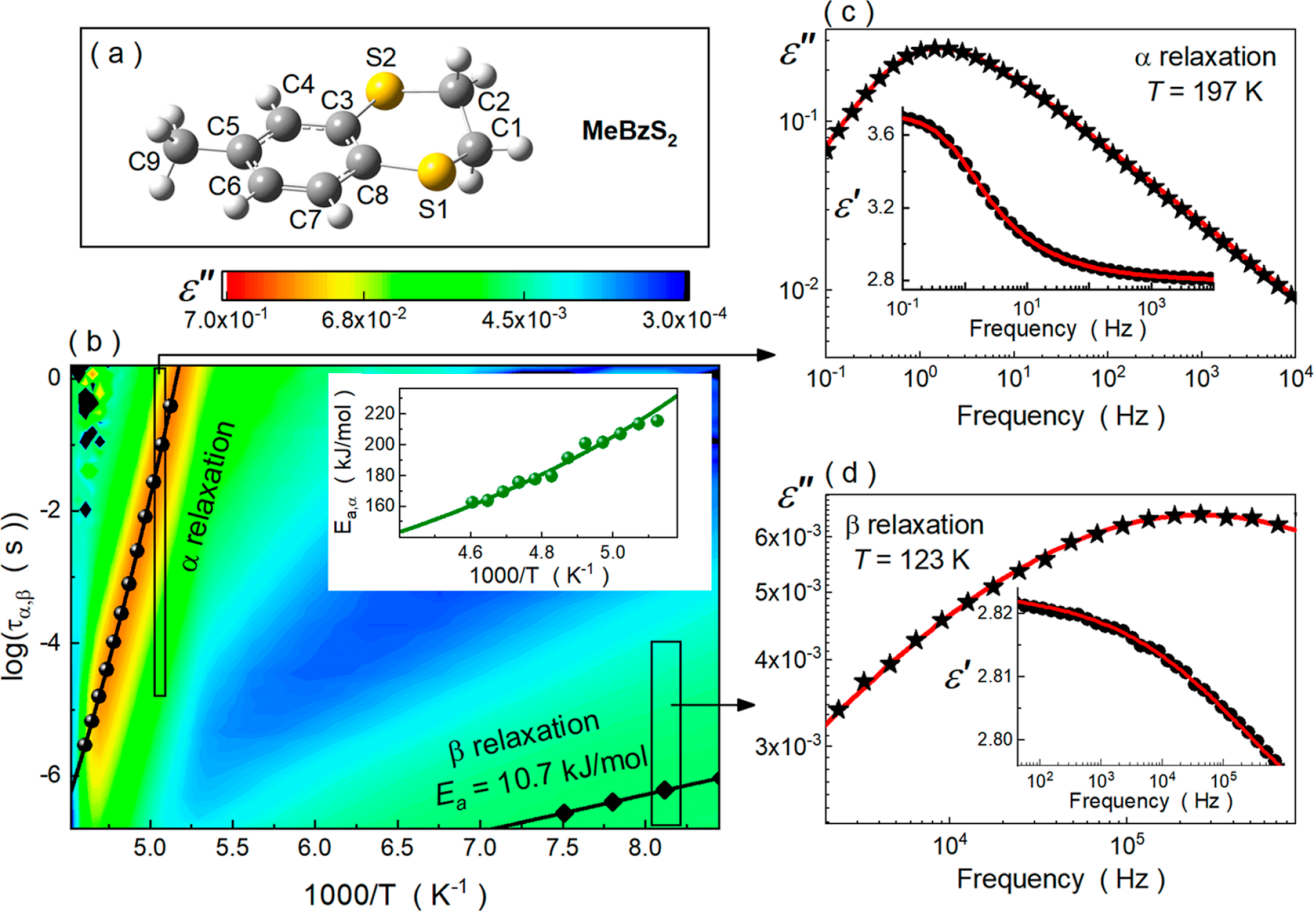
(a) Molecule of MeBzS_2_ with adopted atom labeling. (b) Relaxation times *τ*_*α*_ and *τ_β_* of MeBzS_2_ plotted versus temperature inverse and superimposed on temperature–time dependence of dielectric losses *ε″*. The values of *ε″*are coded by colors. The inset shows temperature-induced changes in activation energy for the α process. (c) Structural α relaxation visible in frequency-dependent *ε″*(*f*) and *ε′*(*f*) spectra measured at 197 K. The red line shows parameterization of experimental data with the Havriliak–Negami function. (d) Representative frequency-dependent *ε″*(*f*) and *ε′*(*f*) spectra measured at 123 K with the apparent secondary β process (black symbols), parameterized with the Cole–Cole function (red line).

A single intramolecular secondary relaxation, labeled as the β process, has also been reported for 6-methyl-2,3-dihydro-1,4-benzodithiine (abbreviated further as MeBzS_2_) – a small heterocycle containing a six-membered thioether ring with two sulfur atoms.^24^ This compound is based on a rigid aromatic toluene-3,4-dithiol building block in which both sulfur atoms are linked by the ethylene bridge into the –S–CH_2_–CH_2_–S– moiety (Figure 1a). Such a moiety constitutes a partial structure of numerous organic donors, e.g., bis(ethylenedithio)tetrathiafulvalene (BEDT-TTF), which is utilized to form superconducting salts.^25−27^ The intramolecular conformational dynamics of the –S–CH_2_–CH_2_–S– motif play a crucial role in determining the physical properties of these materials, including their electronic characteristics or glass-like behavior.^25−27^ MeBzS_2_ itself is a moderately fragile glass-former, displaying low glass transition temperature (*T*_*g*_ = 192 K) and melting point (*T*_*m*_ = 266 K), as well as a considerable tendency toward recrystallization from its supercooled liquid state.^24^ Above its *T*_*g*_, MeBzS_2_ is a conventional van der Waals liquid with negligible intermolecular self-organization tendency.^24^ Consequently, this small heterocycle constitutes a perfect model compound to study the cold crystallization phenomenon, which has not yet been performed on this representative. Regarding the dielectric response, MeBzS_2_ is characterized by two relaxation processes in the dielectric permittivity representation: structural α relaxation and secondary β process.^24^ They have been associated with collective motions of entire molecules in a supercooled liquid and intramolecular conformational transformations within their heterocyclic ring. However, it is essential to note that determining the mechanism behind the β process in MeBzS_2_ has relied solely on a single potential energy curve.^24^ In turn, the unambiguous assignment of secondary relaxation’s mechanism is challenging in the case of heterocyclic compounds, as even in-plane 2D rotational motions should be considered.^28^

In this paper, we revisit molecular dynamics, near-*T*_*g*_ cold crystallization, and relaxation mechanisms of the heterocyclic MeBzS_2_. Following isothermal crystallization kinetics at various temperatures, we uncover a significant interplay between molecular dynamics and cold crystallization from a supercooled liquid. Furthermore, we identify two independent interconversion paths between energetically privileged conformers characterized by strained transition states. We demonstrate that these spatial transformations induce substantial alterations in dipole moment orientation and magnitude. Our investigation also extends to the complex salt PdCl_2_(MeBzS_2_), where we observe a transient conformer directly, revealing a direct relationship between their abundance and the local or macroscopic electric field. Consequently, we confirm the intramolecular character of secondary relaxation in MeBzS_2_ and its mechanism related to conformational changes within the heterocyclic ring. The research is based on the combination of BDS, X-ray diffraction (XRD), and quantum density functional theory (DFT) calculations.

## Experimental Section

### Materials

The object of the studies is an aromatic thioether with a six-membered heterocyclic ring, 6-methyl-2,3-dihydro-1,4-benzodithiine (MeBzS_2_), and its complex salt PdCl_2_(MeBzS_2_). Synthesis, purification, and basic characterization of MeBzS_2_ have been published by us previously.^24^ PdCl_2_(MeBzS_2_) was prepared by dissolving 0.101 mmol (18.5 mg) of MeBzS_2_ and 0.099 mmol (17.8 mg) of PdCl_2_ in 10 mL of CH_3_CN, heating the obtained solution to the boiling point for 20 min, followed by hot filtration. The filtrate was left for slow evaporation in air at an ambient temperature, leading to the formation of orange-red crystals, from which a sample suitable for the XRD study was taken. The obtained solid material was washed with 1 mL of CH_3_CN and dried in air at the ambient temperature, giving an 85% yield (31 mg).

### X-ray Diffraction

The XRD experiment was conducted at 100 K using a SuperNova diffractometer (Agilent Technologies, currently Rigaku Oxford Diffraction) equipped with an Atlas CCD detector and an Oxford Cryosystems cryogenic attachment. The Mo–Kα characteristic radiation (0.71073 Å) was used for the measurements, and data integration was performed using CrysAlis^Pro^ software (v. 1.171.38.41q). The structure of PdCl_2_(MeBzS_2_) was solved with the SHELXS-2013 software via direct methods, and its subsequent refinement was carried out with the SHELXL-2018/3 software.^29^ Hydrogen atoms were treated as riding atoms with U_iso_(H) equal to 1.2U_eq_(C) or 1.5U_eq_(C) and attached in calculated positions. Supplementary crystallographic data for this article are accessible at no cost via the Cambridge Crystallographic Data Centre website under deposition number 2043225.

### Broadband Dielectric Spectroscopy

The BDS technique was performed to reinvestigate the ambient-pressure dielectric response of MeBzS_2_. For this purpose, the sample was poured between two parallel stainless-steel plates of a capacitor, which were 10 mm in diameter and distanced by two quartz fibers of 100 μm thickness. The so-prepared capacitor was sealed by a Teflon ring and cooled to 118 K at a rate of approximately 15 K min^–1^. Dielectric studies were performed under quasi-static conditions between 118 and 221 K, starting from the lowest temperature. Notably, during measurements up to 217 K (i.e., within the temperature at which the relaxation times were determined), the time needed to stabilize and maintain the sample at each temperature was shorter than the time needed to start crystallization, so the sample was kept in the supercooled liquid state. The measurements were performed with steps Δ*T* = 5 K below *T*_*g*_ of MeBzS_2_ or Δ*T* = 2 K for temperatures in the vicinity and above its *T*_*g*_ utilizing the nitrogen gas and a Novocontrol Quattro system for temperature control and stabilization. Dielectric spectra were collected between 10^–1^ and 10^6^ Hz, utilizing a Novocontrol Broadband Dielectric Spectrometer equipped with an Alpha Impedance analyzer.

A different protocol was used, while the crystallization process was monitored with the BDS technique. In this case, the experiment started at room temperature, i.e., above the melting point of MeBzS_2_. In the first step, the liquid was cooled to 153 K (i.e., below its *T*_*g*_) at a rate of roughly 20 K/min and kept at this temperature for 20 min. Subsequently, the sample was heated to the desired temperature at approximately 5 K/min, where the kinetics of isothermal crystallization should be measured. These investigations were performed at 213, 215, 217, 219, 221, 223, and 225 K. The sample was changed after each experiment, and each measurement was repeated twice for each temperature condition to check the results repeatability.

The collected data were subjected to the analysis in the domain of the complex dielectric permittivity: ε* = *ε′* – *iε″*, where ε* is the complex dielectric permittivity, whereas *ε′* and *ε″* are its real and imaginary parts, respectively. The analysis was conducted with WinFit software.

### DFT Calculations

DFT^30,31^ at a hybrid B3PW91 level of theory^32^ and 6-311++G(d,p) basis set^33−35^ was employed to optimize the molecular geometry of MeBzS_2_ with minimum energy (conformer M1). The molecule with adopted atom labeling is presented in Figure 1a. The optimized structure was used as the input file to calculate the vibrational frequency to confirm its identity as an energy minimum. Then, a comprehensive conformational analysis was performed with ±5° step size for five dihedral angles within a heterocycle ring, recording both energy and dipole moment vector variations (see Figure S1 in Supporting Information). All calculations were performed in the gas phase using the Gaussian 16, Revision C.01 software package in a single-molecule approach.^36^

A detailed analysis was conducted for the dihedral angles φ_S1–C1–C2–S2_, φ_C8–S1–C1–C2_, and φ_C1–C2–S2–C3_, which allowed identifying higher-energy saddle conformations T1* and T2*, as well as mutual interconversion between conformers M1 and M2 according to the scheme M1 → [T2*] → M2 → [T1*] → M1. The obtained molecular conformations of MeBzS_2_ at each angle were also utilized for calculating its potential energy surface (PES) defined by dihedral angles φ_S1–C1–C2–S2_ and φ_C8–S1–C1–C2_. Due to the elliptical shape of the PES, the calculation was conducted by determining potential energy curves with a ±5° step size while altering either φ_C8–S1–C1–C2_ with frozen angle φ_S1–C1–C2–S2_ or φ_S1–C1–C2–S2_ with frozen angle φ_C8–S1–C1–C2_. All the collected data were combined to construct a complete PES for MeBzS_2_. All stationary points indicated on the PES were reoptimized using the B3PW91/6-311++G(d,p) level of theory within the Gaussian 16 Revision C.01 software package. Minima and transition state structures were rigorously validated through frequency analyses.

The influence of an oriented external electric field on the geometry and electronic properties of MeBzS_2_ was examined at the same level of theory after prior transforming its geometry to the Z-matrix format according to the previous report.^37^ We utilized the structure of the M1 conformer optimized at the zero field as an input geometry for all the calculations. Notably, the transformation procedure changed its orientation with respect to the *X*, *Y*, and *Z* axes compared to the PES calculations (see Figure S2 in Supporting Information). The oriented external electric field was applied in the Gaussian 16 software along the *X*, *Y,* or *Z* axis by means of the “Field = M ± N” keyword, where M is the direction and N is the field magnitude expressed in atomic units.^37,38^ For example, the notation “Field = X + 10” adds to the Hamiltonian a potential term related to the electric field of 0.001 au (0.514 V/nm)^39^ oriented along the *X* axis.^38^ Under such conditions, the Hamiltonian of a system can be expressed in the first approximation as
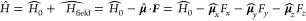
1where *Ĥ*_0_ is the Hamiltonian without an external electric field, *Ĥ*_field_ is a term describing the interaction of a molecule with an electric field, ***μ̂*** is the dipole moment operator, *μ̂*_*x*_, *μ̂*_*y*_, *μ̂*_*z*_ are the *x*, *y*, and *z* components of the dipole moment operator, and ***F*** is the electric field vector with the *F*_*x*_, *F*_*y*_, *F*_*z*_ components.^40−42^ All DFT calculations taking into account of the electric field were performed without any symmetry constraint, utilizing a series of gradients from −0.03 to 0.03 au, which are technically achievable.^38,43^

Finally, we used a hybrid B3PW91 level of theory and diffuse (augmented) functions as in the aug-seg-cc-pVTZ-PP basis set^44,45^ on palladium and 6-311++G(d,p) basis set^33−35^ on sulfur, carbon, chlorine, and hydrogen atoms to dissect various complexation possibilities of PdCl_2_ by the MeBzS_2_ ligand and optimize the possible geometries of the complex salt PdCl_2_(MeBzS_2_). The structure with minimum energy was compared with experimental data and subsequently subjected to modifications within the dihedral angles φ_S1–C1–C2–S2_, φ_C8–S1–C1–C2_, and φ_C1–C2–S2–C3_ to study related energy changes.

## Results and Discussion

Our new broadband dielectric measurements confirm the occurrence of both structural α relaxation and a secondary β process for MeBzS_2_ (Figure 1b–d). Both dielectric relaxations shift toward higher frequencies as the temperature increases, which is related to the progressive shortening of the corresponding relaxation times (*τ_α_*, *τ_β_*) according to the relation
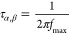
2

In this expression, *f*_max_ is the frequency related to the maximum of the relaxation peak. To determine precisely *f*_max_ (and thus *τ_α_* and *τ_β_*), the α relaxation was parameterized with the Havriliak–Negami function (see Figure 1c).^46^ In contrast, the β process was described with the Cole–Cole function following the previously described procedures (Figure 1d).^24,47^ The relaxation times *τ_α_* and *τ*_*β*_ were calculated from the fit parameters according to the expression
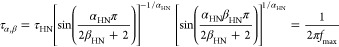
3where *τ_HN_* is the Havriliak–Negami relaxation time and *α_HN_* and *β_HN_* are shape parameters describing the dispersion of dielectric relaxation in the complex dielectric permittivity ε^*^(*f*) domain.^5^ As shown in Figure 1b, *τ_β_* in its logarithm form changes in a linear way with the temperature inverse, obeying the Arrhenius law
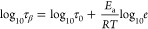
4

In this expression, *E*_a_, *R*, and *τ*_0_ are the activation energy, the gas constant, and the pre-exponential factor determining the relaxation time at the limit of *T* → ∞, respectively. The best fits are obtained when the parameters *E*_a_ and log_10_*τ*_0_ are equal to 10.7 ± 0.5 kJ/mol and −10.7 ± 0.1, respectively, which agrees with the previous study on this compound.^24^ In contrast, the log_10_*τ_α_*(*T*^–1^) curve exhibits a super-Arrhenius character that can be described with the Vogel–Fulcher–Tammann formula
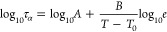
5where *A*, *B*, and *T*_0_ denote the pre-exponential factor, the material constant, and the so-called ideal glass temperature, respectively.^48−50^ In this case, the best fits are obtained for *B*, and *T*_0_ equals to 3540 ± 140 K and 124.2 ± 1.5 K. A characteristic feature of this dependence is that the relaxation peak amplitude starts abruptly decreasing when τ_α_ is close to 3.4 μs, i.e., around 217 K. This phenomenon is well reflected by the temperature–time dependence of *ε″*, which was obtained by transforming the classic *ε″*(*f*, *T*) dielectric spectra into *ε″*(*τ*,1000/*T*) map based on formula 2 and color coding of dielectric losses (Figure 1b). According to the previous report on this compound, the diminishing relaxation peak amplitude corresponds to the cold crystallization of MeBzS_2_ from its supercooled liquid state.^24^ Noteworthily, under these temperature conditions, the apparent activation energy of the α relaxation process, *E*_a*,*α_, ranges between ∼140 and ∼160 kJ/mol (see the inset in Figure 1b). This physical quantity has been determined based on *τ_α_*(*T*^–1^) dependence according to the formula^5^
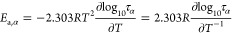
6

To shed more light on the nonequilibrium near-*T*_*g*_ cold crystallization process in MeBzS_2_, we use the BDS technique to study the kinetics of isothermal crystallization between 213 and 225 K.

In general, crystallization is a multistep process, which encompasses the formation of prenucleation aggregates (i.e., self-assemblies of structure similar to the one occurring in the crystal phase), formation of crystal nuclei, and subsequent crystal growth.^6,51,52^ Due to its complex character, crystallization can be affected by a vast array of factors, such as the thermodynamic history of the sample, temperature, or electric field.^6,53^ Therefore, strictly following an adopted experimental protocol for the measurement of crystallization kinetics is essential. In the case of MeBzS_2_, the experiment started at room temperature, i.e., above the melting point of this compound. In the first step, the liquid has been cooled down to 153 K (which is below *T*_*g*_) with a rate of roughly 20 K/min and kept at this temperature for 20 min. Subsequently, the sample was heated at a rate of approximately 5 K/min to the desired temperature, at which the kinetics of isothermal crystallization should be measured. This investigation has been performed at 213, 215, 217, 219, 221, 223, and 225 K with a constant time step. The sample was changed after each experiment, and measurements were repeated twice for each temperature condition to check the results repeatability.

**Figure 2:**
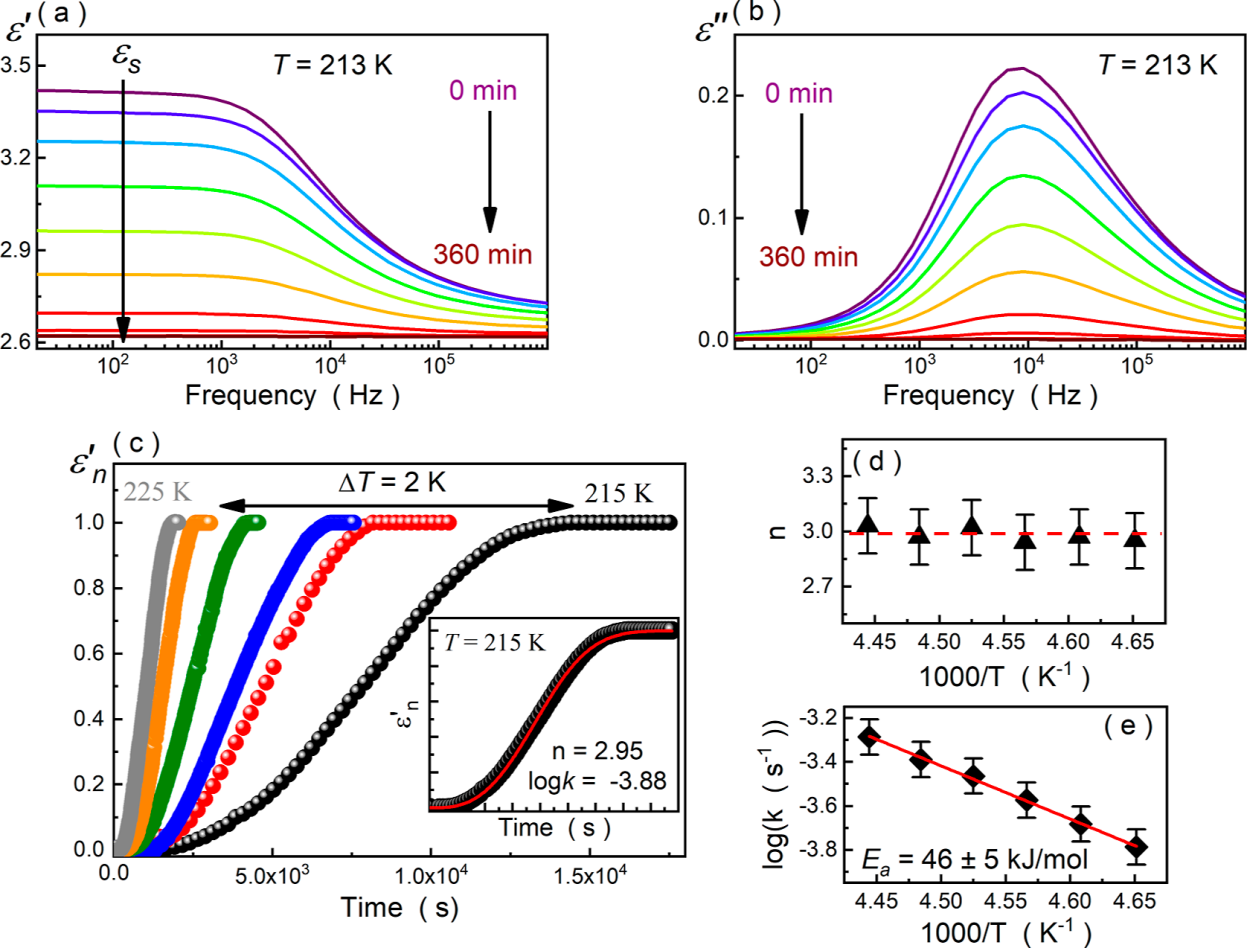
Dielectric *ε′*(*f*) (a) and *ε″*(*f*) (b) spectra registered during isothermal crystallization of MeBzS_2_ at 213 K. (c) Time dependence of the normalized real permittivity during the isothermal crystallization of MeBzS_2_ between 215 and 225 K. (d) Temperature dependence of Avrami exponent *n*. (e) Temperature evolution of crystal growth rate *k* fitted to the Arrhenius law.

Figure 2a,b exhibits representative frequency-dependent dielectric *ε′*(*f*) and *ε″*(*f*) spectra collected while isothermal crystallization of MeBzS_2_ at 213 K. During this process, both static dielectric permittivity (*ε_s_*) and relaxation peak amplitude gradually diminish with time, leading to a complete disappearance of the structural relaxation after approximately 360 min. This phenomenon is related to the increasing crystallinity of the system and, consequently, the decreasing number of relaxing dipoles (molecules) due to their immobilization in the crystal structure
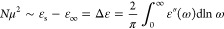
7where *μ* denotes the permanent dipole moments of relaxing molecules and *N* is their total number per unit of volume.^6^ According to this formula, the total vanishing of the structural relaxation is related to 100% conversion of the supercooled liquid to the crystal phase. Moreover, this expression shows that the real and imaginary parts of dielectric permittivity are coupled, and thus, their analysis delivers the same information about dielectric relaxation processes. Hence, to study the kinetics of isothermal crystallization, we will focus solely on the changes in the real part of the dielectric permittivity. In this case, the time-related changes in the crystal volume fraction (*V*_cryst_) concerning the total volume (*V*_total_) of the system are defined in eq. 8
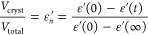
8where *ε′*_*n*_ is the so-called normalized real permittivity, *ε′*(0) is the initial static dielectric permittivity (i.e., the static dielectric permittivity of the liquid phase at given temperature–pressure conditions), *ε′*(*t*) is the static dielectric permittivity at time *t*, and *ε′*(∞) is its value in the long-time limit.^6^Figure 2c depicts the time dependences of *ε′*_*n*_ measured between 215 and 225 K for MeBzS_2_. As can be seen, the crystallization kinetic curves adopt the characteristic S shape independently of the experimental conditions. However, the isothermal crystallization of MeBzS_2_ from its supercooled liquid state slows down with the decreasing temperature from 225 to 215 K. To quantify these changes, we fit the experimental data with the Avrami model
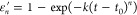
9where *k* is the crystallization rate constant, *t*_0_ is the induction time of crystallization, and *n* is the so-called Avrami exponent, which is related to the dimensionality of the crystallites.^54,55^ In the case of MeBzS_2_, we neglect the variable *t*_0_ (assume that *t*_0_ = 0 s), which has been reported not to introduce any significant error to the analysis if the crystallization of a system is fast.^56^ As shown in the inset of Figure 2c, such an approach is sufficient to describe the experimental data. All parameters characterizing the isothermal crystallization of MeBzS_2_ between 215 and 225 K obtained from the Avrami equation are collected in Table 1.

**Table 1:** Parameters Characterizing the Isothermal Crystallization Kinetics of MeBzS_2_ Obtained from the Avrami Model

temperature (K)	Avrami parameters	
	*k* (s^–1^)	*n*
215	(1.63 ± 0.18)·10^–4^	2.95 ± 0.15
217	(2.08 ± 0.27)·10^–4^	2.97 ± 0.14
219	(2.67 ± 0.13)·10^–4^	2.94 ± 0.15
221	(3.44 ± 0.22)·10^–4^	3.02 ± 0.19
223	(4.08 ± 0.34)·10^–4^	2.97 ± 0.15
225	(5.18 ± 0.45)·10^–4^	3.03 ± 0.12

According to them, the parameter *n* is equal to 3 independently of temperature conditions (Figure 2d). In turn, the characteristic time *τ*_cryst_ decreases when the temperature increases from 215 to 225 K, which corresponds to a gradually growing value of crystallization rate constant *k* in this temperature range (cf. Table 1 and Figure 2e). To dissect this dependence, we parameterize the log_10_*k* = *f*(*T*^–1^) dependence with the Arrhenius law
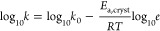
10where *k*_0_ is a fitting parameter and *E*_a,cryst_ is the activation energy for the crystallization process. In the case of MeBzS_2_, *E*_a,cryst_ takes the value of ∼46 kJ/mol, which is approximately three times lower than the activation energy *E*_a,α_ of the structural relaxation process under comparable temperature conditions. Since the ratio between these quantities (*E*_a,cryst_/*E*_a,α_) is precisely the same as the dimensionality of the crystallites (*E*_a,cryst_/*E*_a,α_ = 3 = *n*), it seems plausible that the crystallization process may be controlled (or at least highly affected) by the molecular dynamics in the supercooled liquid.

Apart from translations or reorientations of entire molecules, molecular dynamics of cyclic organic compounds also encompass conformational changes within their structure. Such intramolecular transformations are relevant to MeBzS_2_, and previous study on this compound has predicted two energetically favored conformers and an intermediate transient state.^24^ However, two isomeric transient conformations are possible due to a methyl CH_3_- substituent attached to the aromatic ring. To explore the conformational interconversion possibilities within MeBzS_2_ in more detail, we carried out quantum DFT studies using a single-molecule approach and the 6-311++G(d,p) basis set. Our investigation involved the calculation of potential energy curves for dihedral angles φ_C8–S1–C1–C2_, φ_S1–C1–C2–S2_, and φ_C1–C2–S2–C3_, as well as PES defined by the pair of angles φ_S1–C1–C2–S2_ and φ_C8–S1–C1–C2_.

**Figure 3:**
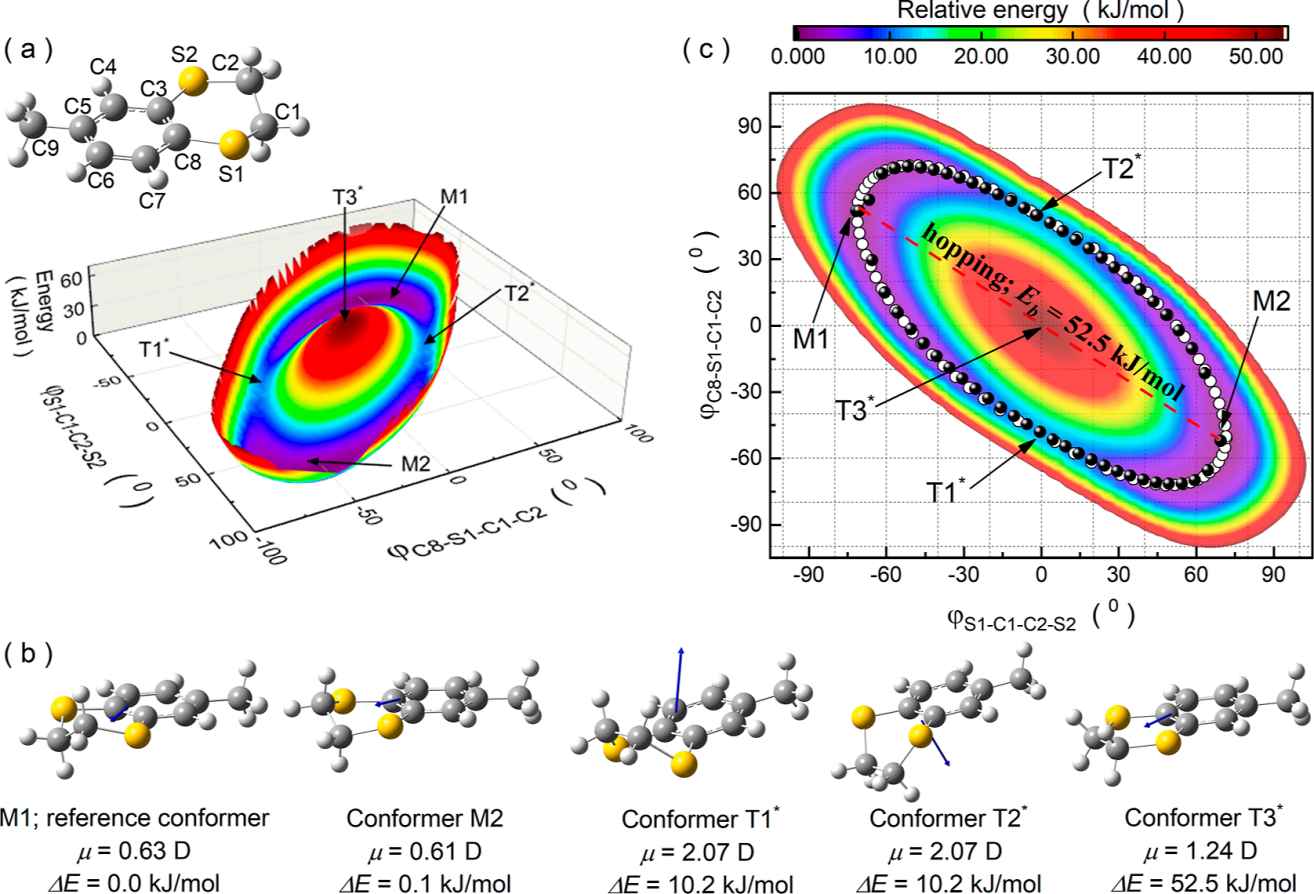
(a) Visualization of the MeBzS_2_ molecule with atom labeling and its associated PES calculated for dihedral angles φ_S1–C1–C2–S2_ and φ_C8–S1–C1–C2_. (b) Geometry of conformers M1, M2, T1*, T2*, and T3* along with their dipole moment vectors. (c) Conformational interconversion paths undergoing according to the direct hopping mechanism (red dashed line) or the sequences M1 →[T1*] → M2, M1 → [T2*] → M2 (black dots). Closed dots donate transformation between conformers M1 and M2 achieved by altering the dihedral angle φ_S1–C1–C2–S2_, while open dots represent the same conformational changes obtained by manipulating dihedral angles φ_C8–S1–C1–C2_ or φ_C1–C2–S2–C3_.

As shown in Figure 3a, the PES of MeBzS_2_ takes the shape of an elliptical double potential well with two energy minima, two saddle points, and a local energy maximum. The two privileged conformers, M1 and M2, are nearly equal in energy. They are characterized by the staggered arrangement of hydrogen atoms within the –CH_2_–CH_2_– bridge (Figure 3b). In this moiety, the carbon atoms are positioned on opposite sides of the aromatic ring plane, resulting in a half-chair geometry and a small dipole moment value of these conformers. In contrast, conformations T1* and T2* corresponding to the saddle points contain eclipsed hydrogen atoms in the –CH_2_–CH_2_– bridge (Figure 3b). Consequently, their defining characteristic is the φ_S1–C1–C2–S2_ dihedral angle, which is close to 0°. This spatial arrangement, featuring two exodentate sulfur atoms, allows for maximizing the dipole moment value. However, due to emerging stresses within the heterocyclic ring, transient conformations T1* and T2* are 10.2 kJ/mol higher in energy than M1 and M2.

**Figure 4:**
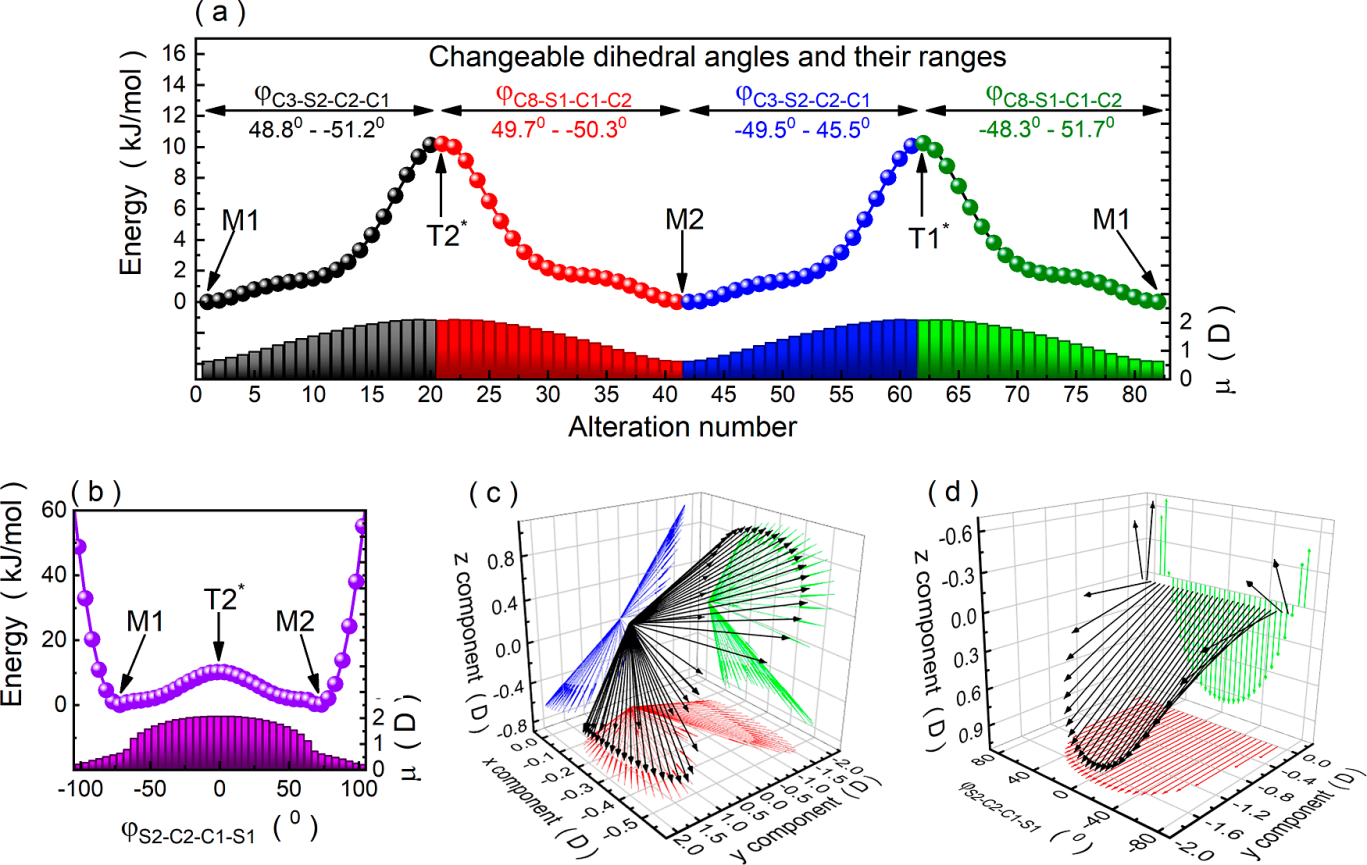
(a) Mutual interconversion between conformers M1 and M2 obtained by altering the dihedral angles φ_C8–S1–C1–C2_ or φ_C1–C2–S2–C3_. (b) Conformational transformation according to the sequence M1 → [T2*] → M2 observed by altering the dihedral angle φ_S1–C1–C2–S2_. (c) Changes in dipole moment orientation in MeBzS_2_ induced by its conformational changes according to schemes M1 → [T2*] → M2 and M1 → [T1*] → M2. (d) Changes in dipole moment orientation (presented for representative *y* and *z* components) and magnitude occurring during mutual interconversion between conformers M1 and M2 following the sequence M1 → [T2*] → M2.

The most energetically disfavored conformation of MeBzS_2_ is T3*. It is roughly 52.5 kJ/mol higher in energy compared to M1 and M2 and features a coplanar alignment of all carbon and sulfur atoms (see Figure 3b). Consequently, the dihedral angles φ_S1–C1–C2–S2_ and φ_C8–S1–C1–C2_ are almost equal to 0°, which makes the structure highly stressed. Noteworthy is that this conformation would appear as a transient geometry during direct interconversion between M1 and M2 according to the hopping mechanism (see Figure 3c). Such a transformation is characterized by an energy barrier of 52.5 kJ/mol, which is much higher than the activation energy of the secondary relaxation process in MeBzS_2_ (10.7 kJ/mol). Consequently, we can exclude direct hopping between conformers M1 and M2 as a source of the β relaxation. The *E*_a_ value fits well to the interconversion path going through T1* or T2* transient state, for which the energy barrier is equal to 10.2 kJ/mol (cf. Figures 1b and 3b). According to calculations, these conformational transformations can be achieved by altering not only the dihedral angle φ_S1–C1–C2–S2_ (as reported previously) but also φ_C8–S1–C1–C2_ or φ_C1–C2–S2–C3_ (see Figure 4a,b). In fact, in the case of this interconversion path, the dihedral angles φ_C8–S1–C1–C2_ or φ_C1–C2–S2–C3_ are related to φ_S1–C1–C2–S2_ by the formula of the rotated ellipse
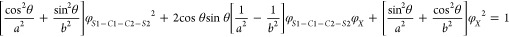
11where φ_*X*_ = φ_C8–S1–C1–C2_ or φ_C1–C2–S2–C3_ and parameters *a*, *b*, and *θ* are equal to 38.6° ± 0.2°, 93.2° ± 0.2°, and 0.79 ± 0.01, respectively. It is worth highlighting that conformational changes following the sequences M1 → [T1*] → M2 and M1 → [T2*] → M2 bring about a substantial alteration in both the spatial orientation and the magnitude of the dipole moment vector of MeBzS_2_ (Figure 4c,d). Even minor adjustments in dihedral angle φ_S1–C1–C2–S2_ considerably influence the dipole moment vector (Figure 4d). The close correspondence between the energy barrier and *E*_a_, coupled with the substantial potential for spatial rearrangement of the dipole moment vector and significant fluctuations in its magnitude, strongly suggests that this mode of conformational interconversion represents the most likely source of intramolecular β relaxation in MeBzS_2_. To validate this hypothesis, we investigate the behavior of MeBzS_2_ when exposed to an external electric field.

**Figure 5:**
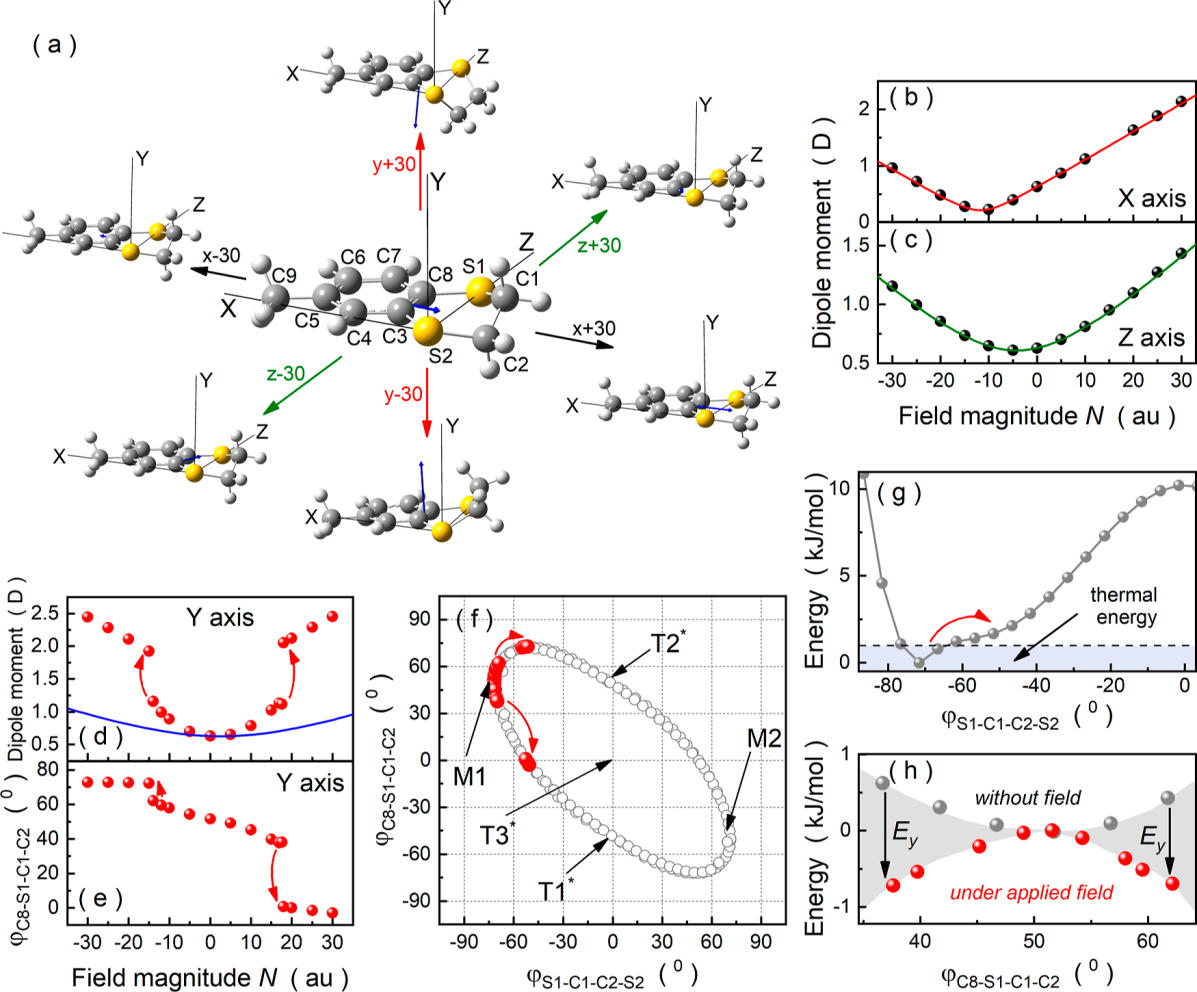
(a) Orientation of the MeBzS_2_ molecule in the Cartesian coordinate system as well as field-induced changes in the MeBzS_2_ geometry and its dipole moment vector orientation. (b) Changes in the dipole moment of MeBzS_2_ under the applied electric field oriented along the *X* axis (black dots) and related fits according to expression (13). (c) Total dipole moment of MeBzS_2_ versus the applied electric field oriented along the *Z* axis (black dots) fitted with function (13). (d) Field-induced changes in the dipole moment of MeBzS_2_ related to the induced dipole moment (blue curve) and conformational changes (red dots) when applying the electric field along the *Y* axis. (e) Field-induced changes in the dihedral angle φ_C8–S1–C1–C2_ of MeBzS_2_ when exposed to the electric field oriented along the *Y* direction. (f) Field-induced conformational interconversion path (red dots) superimposed on the sequence M1 → [T1*] → M2 → [T2*] → M1. (g) Possible conformational changes in MeBzS_2_ under low electric fields. (h) Stabilization of transient conformers of MeBzS_2_ under an external electric field oriented along the *Y* direction.

First, we examine the response of the MeBzS_2_ molecule to an oriented electric field using DFT calculations. For this purpose, we align conformer M1 so that its aromatic ring lies almost in the *XZ* plane and the sulfur atoms are aligned along the *Z* axis. As shown in Figure 5a, applying an electric field along the *X* or *Z* direction to such an oriented molecule does not change its conformation significantly, altering its bond lengths, angles, and torsion angles to a small extent. Only the dipole moment considerably changes its direction and magnitude with the electric field in this case (see Figure 5a,b,c). This phenomenon originates from the nonzero polarizability and hyperpolarizability tensors of MeBzS_2_ and, consequently, the appearing induced dipole moment. Namely, the field dependence of the dipole moment vector in polar systems is described in eq. 12
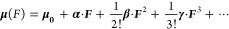
12where ***μ***_**0**_ is the permanent (inherent) dipole moment vector, ***α*** is the molecular polarizability tensor, and ***β*** and ***γ*** are the second- and third-order hyperpolarizabilities.^42^ The symbol ***F*** represents the external electric field vector.^42^ As indicated with the red line in Figure 5b and the green curve in Figure 5c, the field dependence of the dipole moment value in MeBzS_2_ exposed to the electric field oriented along the *X* or *Z* direction can be sufficiently described by the following approximation of eq. 12, in which the hyperpolarizability tensors are neglected
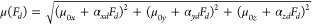
13where *d* = *x* or *z* denotes the specific direction (*X* or *Z*), *μ*_0*x*_ = −0.5974D, *μ*_0*y*_ = 0.0688D, and *μ*_0*z*_ = −0.1825D are the *x*, *y*, and *z* components of the permanent dipole moment vector determined for the optimized zero-field geometry M1, whereas α_*xx*_ = 32.4384 × 10^–40^ C^2^m^2^J^–1^, α_*yx*_ = 0.1635 × 10^–40^ C^2^m^2^J^–1^, α_*xx*_ = −1.0863 × 10^–40^ C^2^m^2^J^–1^, α_*xz*_ = −1.0863 × 10^–40^ C^2^m^2^J^–1^, α_*yz*_ = 0.1601 × 10^–40^ C^2^m^2^J^–1^, and α_*zz*_ = 24.0676 × 10^–40^ C^2^m^2^J^–1^ are the components of the polarizability matrix of the optimized zero-field structure M1.

Significant changes in the dipole moment value also occur when the electric field is applied along the *Y* axis on conformer M1 (Figure 5d). However, in this case, these changes result primarily from field-induced modifications in the geometry of MeBzS_2_ (cf. Figure 5a,d). Namely, as shown in Figure 5d, there is a significant discrepancy between the calculated dipole moment values (red dots in Figure 5d) and predictions made according to eq. 13 with *d* = *y* and the following components of the polarizability matrix of the optimized zero-field structure M1: α_*xy*_ = 0.1635 × 10^–40^ C^2^m^2^J^–1^, α_*yy*_ = 14.5925 × 10^–40^ C^2^m^2^J^–1^, and α_*zy*_ = 0.1611 × 10^–40^ C^2^m^2^J^–1^ (blue line in Figure 5d). The DFT calculations suggest that even small electric fields along the *Y* axis can induce conformational transformations. For instance, applying the oriented electric field along the −*Y*-axis direction causes the dihedral angle φ_C8–S1–C1–C2_ to increase nonlinearly to roughly 73° (Figure 5e). In turn, increasing the electric field along the +*Y*-axis direction up to 3 × 10^–3^ au (1.542 V/nm) gradually decreases this dihedral angle value to approximately −2.8°. Notably, the geometry of MeBzS_2_ deviates significantly from a half-chair conformation below −1.5 × 10^–3^ au (0.771 V/nm) and above +1.8 × 10^–3^ au (0.925 V/nm) when the electric field is applied along the *Y* axis. As illustrated in Figure 5f, the field-induced conformational changes in MeBzS_2_ closely resemble those observed for the interconversion path M1 → [T2*] → M2 → [T1*] → M1, particularly in terms of the variation in dihedral angles within the heterocyclic moiety. It means that if the entire molecules are frozen, as in the glass phase, the orientational polarization may indeed arise from conformational changes following this interconversion path. This statement is also reinforced when concerning the thermal energy delivered to the condensed system, which does not exceed 1.6 kJ/mol for the glass phase (*T* < *T*_*g*_ = 192 K). This value is sufficient to change the dihedral angles φ_S1–C1–C2–S2_ and φ_C8–S1–C1–C2_ to approximately −65 and 57°, respectively (Figure 5g). According to the DFT calculations, it is possible to change the conformation of MeBzS_2_ significantly under such conditions by applying electric fields not exceeding 1 × 10^–5^ au (i.e., less than 5.14 × 10^6^ V/m), which are easily applicable in the dielectric spectroscopy technique (see the red arrow in Figure 5d,e,f,g).^57^

What is more, transient conformers are energetically favored over conformer M1 in the presence of an external electric field oriented along the *Y* axis (see Figure 5h). This feature can be explained by utilizing the Taylor expansion of the molecular system energy under an electric field^42^
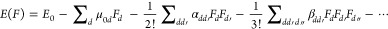
14where *d*, *d′*, and *d′′* denote the specific components (*x*, *y*, or *z*) of the corresponding vectors or tensors. According to this expression, the energy *E*_0_ of a specific conformer becomes corrected under the electric field *F* by terms containing permanent dipole moment *μ*_0_, polarizability *α*, and hyperpolarizabilities *β*.^42^ Minimizing *E* at a specific electric field *F* requires maximization of the *μ*_0_ value and collinear alignment of ***μ***_**0**_ and ***F*** vectors. Maximizing *μ*_0_ can be achieved by transforming MeBzS_2_ into the strained intermediate conformation T1* or T2*. Consequently, one might even expect field-induced induction of the transient geometries T1* and T2* for a specific alignment of MeBzS_2_ molecules with respect to the electric field lines. One can conclude that the substantial potential for temperature- and field-induced spatial rearrangement of the dipole moment vector in MeBzS_2_, the related significant fluctuations in its magnitude, the ease of field-induced conformational changes within the heterocyclic ring, the field-induced stabilization of transient geometries, and the previously mentioned close correspondence between the energy barrier and activation energy *E*_a_ clearly indicate that the interconversions following the scheme M1 → [T2*] → M2 → [T1*] → M1 indeed occur in the MeBzS_2_ and are responsible for its secondary β relaxation. To support experimentally our theoretical predictions, we investigate the behavior of MeBzS_2_ in the presence of metal cations and synthesize the complex salt PdCl_2_(MeBzS_2_).

**Figure 6:**
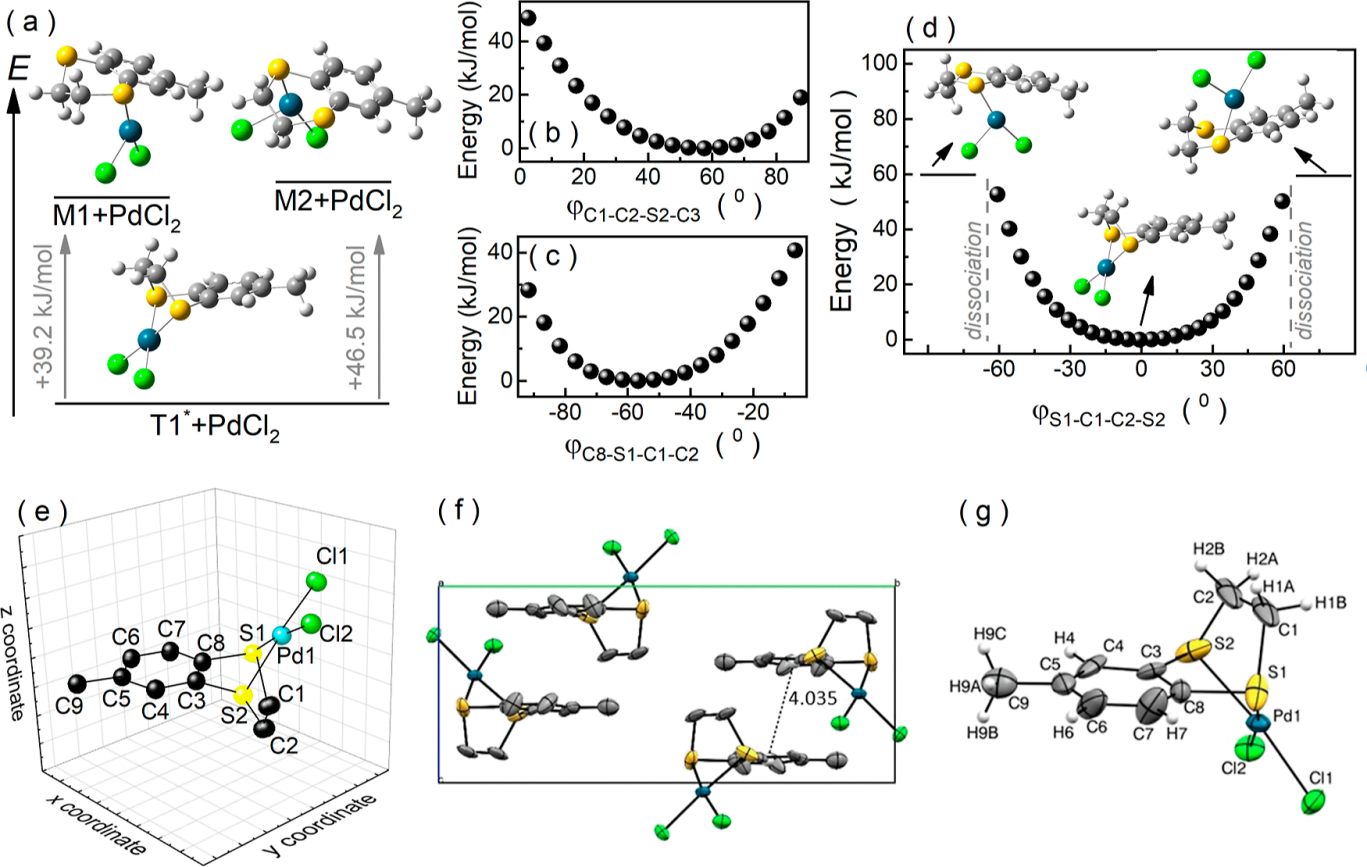
(a) Different complexation possibilities of PdCl_2_ by the MeBzS_2_ molecule compared in terms of the energy of the formed structures. (b) Changes in the energy of PdCl_2_(MeBzS_2_) observed while altering the dihedral angle φ_C1–C2–S2–C3_. (c) Variation of PdCl_2_(MeBzS_2_) energy while changing the dihedral angle φ_C8–S1–C1–C2_. (d) Energy and structural modifications in complex salt PdCl_2_(MeBzS_2_) resulting from changes in the dihedral angle φ_S1–C1–C2–S2_. (e) Visualization of possible displacements of the nonhydrogen atoms in PdCl_2_(MeBzS_2_) induced by the thermal energy of ∼0.83 kJ/mol. (f) Packing diagram of PdCl_2_(MeBzS_2_) along the crystallographic axis *a*, highlighting distances between centroids. (g) The asymmetric unit with adopted atom numbering.

In general, the complexation of metal cations by organic ligands can occur in a solution. Under these conditions, the unreacted free chemical individua remain in thermal equilibrium with the formed complex salts of various geometries, which is quantified by a stability constant.^58^ According to the DFT calculations, the complexation of Pd^2+^ by MeBzS_2_ in geometries close to conformers M1 or M2 is ineffective and leads to unstable higher-energy structures (Figure 6a). In turn, transient conformations T1* and T2* are the most energetically privileged geometries of MeBzS_2_ in the presence of PdCl_2_ (Figure 6a). Their preferred occurrence can be ascribed, among others, to charge–dipole interactions. Namely, ions are the source of the local electric field, and any polar objects (i.e., possessing a permanent electric dipole moment) are subject to torque when placed in an external electric field.^59,60^ The torque makes the dipoles align parallel with the field so that the potential energy is minimized and the attractive Coulomb interactions with ionic species are the most effective. Eq. 15 defines the standard Coulomb energy describing the charge–dipole interaction
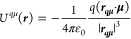
15where ε_0_ is the vacuum permittivity, *q* is the charge on an ion, ***μ*** is the dipole moment of an organic molecule, and |*r*_*q*μ_| is the center of mass distance between the charge and the molecular dipole.^59^ Interaction between Pd^2+^ and lone pairs of S atoms from MeBzS_2_ is ineffective for conformers M1 and M2 because of the considerable angle between vectors ***r***_***qu***_ and ***μ***. Maximizing the Coulomb energy (in terms of its absolute value) requires transforming MeBzS_2_ to the strained intermediate conformer T1* (or T2*), characterized by the highest dipole moment value and almost collinear alignment of vectors ***r***_***qu***_ and ***μ***. Considering the attractive nature of the Pd^2+^–MeBzS_2_ interactions, the entire energy of the forming PdCl_2_(MeBzS_2_) is minimized in this way. Indeed, as shown in Figure 6b–d, any deviation from conformation T1* (or T2*) increases the energy of the complex salt PdCl_2_(MeBzS_2_) by several dozens of kJ/mol. Notably, the complexation of Pd^2+^ cations hampers the intramolecular conformational dynamics of the –S–CH_2_–CH_2_–S– motif in MeBzS_2_, which is a different scenario from the one observed for BEDT-TTF and its superconducting salts.^25−27^ For example, shifting the value of dihedral angle φ_S1–C1–C2–S2_ from ∼0° to ∼ −60° or ∼60° increases the energy of PdCl_2_(MeBzS_2_) so much that PdCl_2_ dissociates and binds to only one sulfur of MeBzS_2_ (Figure 6d). Consequently, only small structural modifications are possible within the heterocyclic ring of MeBzS_2_. For instance, at 100 K, when the thermal energy delivered to the system is roughly 0.83 kJ/mol, the dihedral angles φ_C1–C2–S2–C3_, φ_C8–S1–C1–C2_, and φ_C8–S1–C1–C2_ may vary within the following ranges: φ_C1–C2–S2–C3_ ∈ (48.7°, 65.9°), φ_C8–S1–C1–C2_ ∈ (−65.0°, −47.8°), and φ_C8–S1–C1–C2_ ∈ (−12.5°, 11.4°). These possible angular fluctuations correspond to small displacement of atoms S1, S2, C1, and C2, which do not exceed 0.03, 0.03, 0.10, and 0.09 Å, respectively (Figure 6e). Consequently, one can expect a well-ordered structure of the complex salt PdCl_2_(MeBzS_2_) in its crystal phase with relatively small thermal ellipsoids. To prove this hypothesis, we performed XRD measurements.

Complex salt PdCl_2_(MeBzS_2_) crystallizes in a monoclinic system with space group *P*2_1_/*c* (see Table S1 for more details). As depicted in Figure 6f, the unit cell contains four molecules of the salt aligned in a way that allows the formation of intermolecular interactions and Cl···H. The distance between centroids of interacting benzene rings via π···π stacking is equal to 4.035 Å. In turn, the Cl···H distances across their short contracts take the following values: 2.790 Å for Cl1···H7(1–*x*, 1–*y*, −*z*), 2.930 Å for Cl1···H9C(*x*, 1.5–*y*, 1/2 + *z*), 2.850 Å for Cl1···H9B(1–*x*, −1/2 + *y*, 1/2–*z*), 2.693 Å for Cl1···H2A(−*x*, 1–*y*, −*z*), 2.781 Å for Cl1···H1A(*x*, *y*, 1 + *z*), 2.801 Å for Cl2···H1B(−*x*, 1–*y*, −*z*), 2.932 Å for Cl2···H4(*x*, 1.5–*y*, 1/2 + *z*), and 2.837 Å for Cl2···H9A(−1 + *x*, 1.5–*y*, 1/2 + *z*).

Each Pd^2+^ cation (Pd1) in the complex salt is coordinated by two sulfur atoms, S1 and S2, of a single heterocyclic organic ligand and two chlorine ions, Cl1 and Cl2 (Figure 6g). The distances between Pd^2+^ cation and the coordinating individua S1, S2, Cl1, and Cl2 are equal to 2.261(3), 2.286(3), 2.293(3), and 2.306(3) Å, respectively. The heterocyclic organic ligand MeBzS_2_ adopts a highly stressed and energetically unfavorable conformation in its complex salt with the opposite conformation of hydrogen atoms, and both carbon atoms of the ethylene bridge –CH_2_–CH_2_– are located on the same side of the aromatic ring plane. Noteworthy is that this architecture closely resembles the geometry of the transition state T1*, which agrees with previous predictions made by DFT calculations. The value of the dihedral angle φ_S1–C1–C2–S2_ deviates from 0° to a small extent, being equal to 7(1)°. This value falls within the range of (−12.5°, 11.4°), which is previously predicted by DFT. It also agrees with the anticipated small atom displacements with respect to the conformer T1*. Consequently, it is likely that the observed small deviation from T1* geometry results from thermal fluctuations. However, intermolecular interactions occurring in the crystal structure should also be taken into account.

The direct observation of the transient geometry close to T1* supports our previous predictions that conformational changes in MeBzS_2_ can occur according to the scheme M1 → [T2*] → M2 → [T1*] → M1. It also reinforces our conclusion that the β relaxation of MeBzS_2_ is related to the intramolecular conformational dynamics (not an in-plane rotation of molecules). The initially energetically disfavored transient geometries become energetically privileged after the electric field is applied or in the presence of ions. This phenomenon may even induce an unprecedented inversion of states in which all of the MeBzS_2_ molecules are transformed from the M1 or M2 geometries to the transient conformer T1* or T2*, as observed in the crystal structure of PdCl_2_(MeBzS_2_).

## Conclusions

MeBzS_2_ is a moderately fragile heterocyclic glass-former with a considerable tendency toward crystallization from the supercooled liquid state. Its cold crystallization is characterized by activation energy equal to 46 ± 5 kJ/mol and is profoundly influenced by molecular dynamics. In terms of dielectric response, this heterocycle is characterized by the structural α relaxation and a secondary β process, which offer insights into dynamics on various molecular scales. The first process is associated with cooperative reorientations of entire molecules in the supercooled liquid. In turn, the dielectric β relaxation has intramolecular character for MeBzS_2_, originating from mutual interconversions between two energetically favored conformers with half-chair geometries (M1 and M2). Notably, these conformational changes do not adhere to a direct hopping mechanism between these conformers. Instead, they proceed through two transient geometries, T1* and T2*, corresponding to the saddle points of the elliptical-shaped PES of MeBzS_2_. These intramolecular transformations induce substantial alterations in both dipole moment orientation and magnitude. The highest dipole moment value is observed for the transient conformers, which contain eclipsed hydrogen atoms in the –CH_2_–CH_2_– bridge and two exodentate sulfur atoms. Consequently, the initially energetically privileged conformations M1 and M2 in an undisturbed system become less favored after the electric field is applied or in the presence of ions. This phenomenon may even lead to an unprecedented inversion of states in which all of the MeBzS_2_ molecules are transformed to the transient conformer T1* or T2*. The intriguing behavior has been mathematically rationalized and confirmed by the crystal structure of the complex salt PdCl_2_(MeBzS_2_), in which the transient conformer has been directly observed.

## Supporting Information Available

Crystal structure of PdCl_2_(MeBzS_2_) and DFT calculations of MeBzS_2_ (PDF)
Nowok_structure_MeBzS2 (CIF)

## Supplementary Material

jp3c06727_si_001.pdf

jp3c06727_si_002.cif
