# Supplementary material for: Revisiting Dynamic Processes and Relaxation Mechanisms in a Heterocyclic Glass-Former: Direct Observation of a Transient State
Source: J Phys Chem B. 2024 Feb 21;128(8):1915–26. doi: 10.1021/acs.jpcb.3c06727 (PMC10910497; doi:10.1021/acs.jpcb.3c06727)
Supplement: Supplementary file 1 — jp3c06727_si_001.pdf [file jp3c06727_si_001.pdf]

Revisiting Dynamic Processes and Relaxation Mechanisms in a Heterocyclic Glass-Former: Direct Observation of a Transient State

Supplementary Materials

Andrzej Nowok[1,2,*], Hubert Hellwig[3], Mateusz Dulski[4], Maria Książek[5], Joachim Kusz[5], Piotr Kuś[6] and Sebastian Pawlus[5]

[1] *Department of Experimental Physics, Wrocław University of Science and Technology, Wybrzeże Wyspiańskiego 27, 50-370, Wrocław, Poland;*

[2] *Laboratoire National des Champs Magnétiques Intenses, EMFL, CNRS UPR 3228, Université Toulouse, Université Toulouse 3, INSA-T, Toulouse 31400, France;*

[3] *Center for Integrated Technology and Organic Synthesis (CiTOS), MolSys Research Unit, University of Liège, B6a, Room 3/19, Allée du Six Août 13, 4000 Liège, Sart Tilman, Belgium*

[4] *Faculty of Science and Technology, Institute of Materials Engineering, University of Silesia in Katowice, 75 Pułku Piechoty 1A, 41-500 Chorzów, Poland*

[5] *August Chełkowski Institute of Physics, University of Silesia in Katowice, 75 Pułku Piechoty 1, 41-500 Chorzów, Poland;*

[6] *Institute of Chemistry, University of Silesia in Katowice, Szkolna 9, 40-003 Katowice, Poland;*

*andrzej.nowok@pwr.edu.pl

1. Additional information for DFT calculation

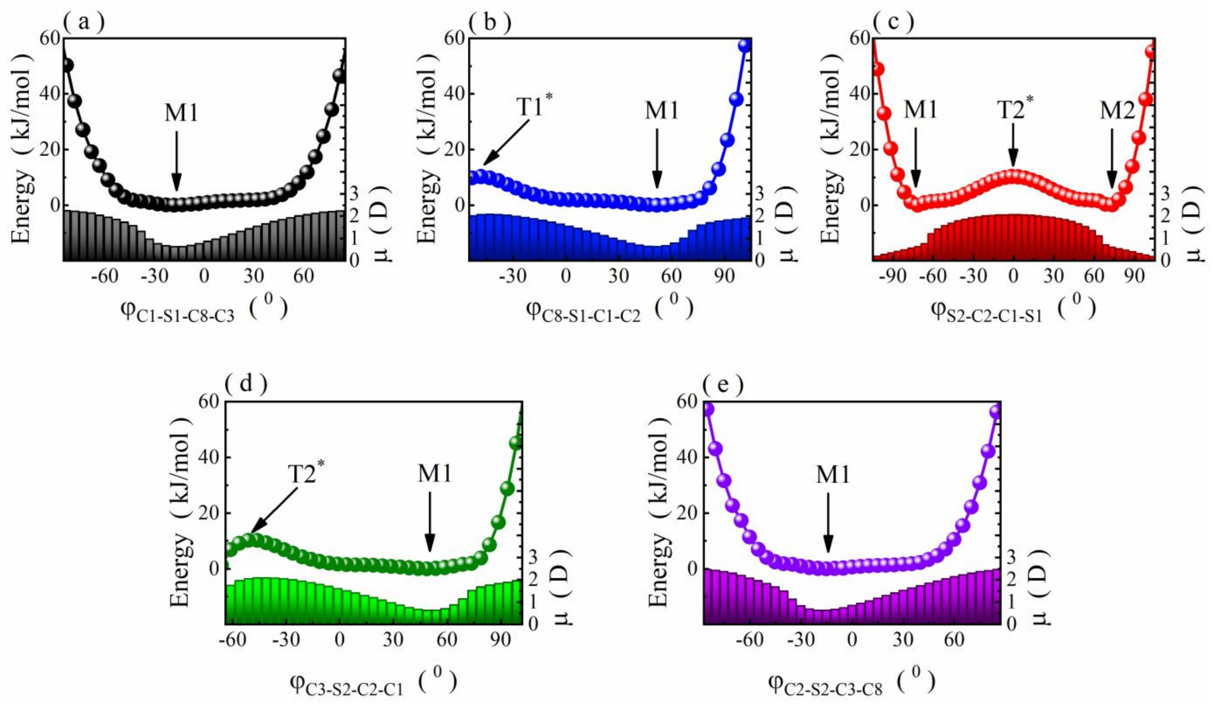

Figure S1. Potential energy curves for $MeBzS_2$ (color dots) and related changes in dipole moment value (color bars) obtained while altering the dihedral angles (a) $\varphi_{C1\text{-}S2\text{-}C8\text{-}C3}$, (b) $\varphi_{C8\text{-}S1\text{-}C1\text{-}C2}$, (c) $\varphi_{S2\text{-}C2\text{-}C1\text{-}S1}$, (d) $\varphi_{C3\text{-}S2\text{-}C2\text{-}C1}$, and (e) $\varphi_{C3\text{-}S2\text{-}C2\text{-}C1}$. The potential energy was calculated concerning conformer M1, and its geometry was used as a starting point for the computation.

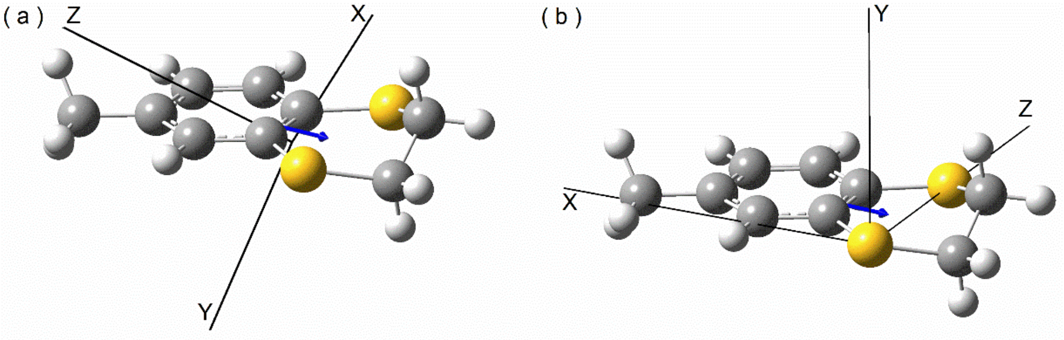

Figure S2. Orientation of M1 conformer in the Cartesian coordinate system used while (a) calculating potential energy surface and curves, and (b) modelling the influence of an oriented external electric field.

2. Additional information for crystal structure of $PdCl_2(MeBzS_2)$

Table S1. Crystallographic data for crystal structure of $PdCl_2(MeBzS_2)$.

T/K	100
CCDC number	2043225
Chemical formula	$C_9H_{10}Cl_2PdS_2$
Formula Mass	359.59
Crystal system	Monoclinic
Space group	P 2_1/c
Z	4
Unit cell dimensions<br>*a*/Å<br>*b*/Å<br>*c*/Å<br>*β*/°	 8.5099(6)<br>17.7541(9)<br>7.7967(5)<br>101.724(7)
Unit cell volume/Å^3	1153.39(13)
F(000)	704
D_x (Mg m^{-3})	2.071
μ (mm^{-1})	2.389
Theta range for data collection/^{0}	3.353 to 26.365
Completeness	theta = 25.242°<br>99.8 %
Range of *h*, *k*, *l*	$-10 \leq h \leq 10$<br>$-22 \leq k \leq 14$<br>$-9 \leq l \leq 9$
No. of measured reflections	9388
No. of independent reflections	2359
R_{int}	0.0614
Data / restraints / parameters	2359 / 0 / 122
Goodness-of-fit on F^2	1.170
Final R_1 values ($I > 2\sigma(I)$)	0.0817
Final $wR(F^2)$ values ($I > 2\sigma(I)$)	0.1716
Final R_1 values (all data)	0.0967
Final $wR(F^2)$ values (all data)	0.1793
Largest diff. peak and hole/eÅ^3	2.289 and -1.578
